# Population Genomics Refines Population Structure and Individual Stock Assignment in Atlantic and Mediterranean Swordfish (*Xiphias gladius*)

**DOI:** 10.3390/ijms27198612

**Published:** 2026-09-26

**Authors:** Giorgia Gioacchini, Kyle Gillespie, Daniela Rosa, Sara Filippi, Luca Marisaldi, Dheeraj Busawon, Natalia Bezerra, Paulo Travassos, Hilário Tiago Campello, Fábio Hissa Vieira Hazin, David Macías, Alexia Gallucci, Francois Poisson, Fulvio Garibaldi, Luis Gustavo Cardoso, Luigi Pappalardo, Paolo Pignalosa, Riccardo Aiese Cigliano, Su Nan-Jay, Dan Parker, George Tserpes, Freddy Arocha, Taimi Shikongo, Tommaso Righi, Andrea Splendiani, Antonio Di Natale, Piero Addis, Adriano Mariani, Miguel Neves dos Santos, Rui Coelho, Alex Hanke, Oliana Carnevali

**Affiliations:** 1 Dipartimento di Scienze della Vita e dell’Ambiente, Universita’ Politecnica delle Marche, 60131 Ancona, Italy; s.filippi@univpm.it (S.F.); l.marisaldi@univpm.it (L.M.); tommaso.righi@univpm.it (T.R.); andrea.splendiani@univpm.it (A.S.); 2 Fisheries and Oceans Canada, St. Andrews Biological Station, 125 Marine Science Drive, St. Andrews, NB E5B 0E4, Canada; kyle.gillespie@dfo-mpo.gc.ca (K.G.); dheeraj.busawon@dfo-mpo.gc.ca (D.B.); alex.hanke@dfo-mpo.gc.ca (A.H.); 3 Instituto Português do Mar e da Atmosfera (IPMA), Av. 5 de Outubro s/n, 8700-305 Olhão, Portugal; dfrosa@ualg.pt (D.R.); rpcoelho@ualg.pt (R.C.); 4 Centro de Ciências do Mar da Universidade do Algarve (CCMar), Universidade do Algarve, Campus de Gambelas, 8005-139 Faro, Portugal; 5 Instituto de Oceanografia, Universidade Federal Rural de Pernambuco, Av. Dom Manoel de Medeiros, s/n Dois Irmãos, 52171-900 Recife, PE, Brazil; natalia_pab@hotmail.com (N.B.); paulo.travassos@ufrpe.br (P.T.); tiagohpcampello@ufrpe.br (H.T.C.);; 6 Centro Oceanográfico de Málaga, Instituto Español de Oceanografía (IEO-CSIC), Puerto Pesquero s/n, 29640 Fuengirola, Spain; david.macias@ieo.csic.es; 7 AquaBioTech Group, Central Complex, Naggar Street, Targa Gap, MST 1761 Mosta, Malta; amg@aquabt.com; 8 MARBEC, Université de Montpellier, CNRS, Ifremer, IRD, 34203 Sète, France; francois.poisson@ifremer.fr; 9 Department of Earth, Environment and Life Sciences, University of Genoa, Corso Europa, 26, 16132 Genova, Italy; largepel@unige.it; 10 Laboratório de Recursos Pesqueiros Demersais e Cefalópodes, Instituto de Oceanografia, Universidade Federal do Rio Grande, Av. Itália, sn, Campus Carreiros, 96203-900 Rio Grande, RS, Brazil; cardosolg15@gmail.com; 11 OCEANIS SRL, 80056 Ercolano, Italy; oceanissrl1@gmail.com (L.P.); oceanissrl@gmail.com (P.P.); 12 Sequentia Biotech SL, 08005 Barcellona, Spain; raiesecigliano@sequentiabiotech.com; 13 Department of Environmental Biology and Fishery Science, National Taiwan Ocean University, Keelung 20224, Taiwan; nanjay@ntou.edu.tw; 14 Department of Biological Sciences, University of Cape Town, Rondebosch, Cape Town 7700, South Africa; dparker@dffe.gov.za; 15 Hellenic Centre for Marine Research, Athens Sounio Ave., P.O. Box 712, Anavyssos, 19013 Attiki, Greece; gtserpes@hcmr.gr; 16 Instituto Oceanográfico de Venezuela, Universidad de Oriente, A.P. 204, 6101 Cumana, Venezuela; farochap@gmail.com; 17 Ministry of Fisheries and Marine Resources, National Marine Information and Research Centre, Swakopmund P.O. Box 912, Namibia; taimi.shikongo@mfmr.gov.na; 18 Aquastudio Research Institute, Via Trapani, 6, 98121 Messina, Italy; adinatale@costaedutainment.it; 19 Department of Life Science and Environment, University of Cagliari, Via Fiorelli 1, 09126 Cagliari, Italy; addisp@unica.it; 20 UNIMAR Società Cooperative, Via Nazionale, 243, 00184 Roma, Italy; a.mariani@unimar.it; 21 ICCAT Secretariat, Calle Corazón de Maria 8, Planta 6, 28002 Madrid, Spain; miguel.santos@iccat.int

**Keywords:** reference genome, ddRAD sequencing, genetic differentiation, fisheries management, genetic diversity, genetic monitoring

## Abstract

Swordfish (*Xiphias gladius*) is managed in the Atlantic Ocean and Mediterranean Sea as three major stocks, although their correspondence with underlying genomic population structure remains uncertain. Here, we combined a newly assembled reference genome with ddRAD sequencing of 575 swordfish from 14 Atlantic and Mediterranean areas to characterize genomic population structure and genetic differentiation relative to the current ICCAT management framework, evaluate individual stock assignment and ancestry across management boundaries, and compare genetic-diversity patterns among stocks and sampling areas. After quality control, 504 individuals were retained, with 26,324 SNPs used for diversity analyses and 15,049 LD-pruned SNPs for population-structure and assignment analyses. Multivariate analyses, pairwise differentiation, AMOVA, and ADMIXTURE consistently identified strong Mediterranean–Atlantic differentiation, whereas North and South Atlantic swordfish showed much weaker and partly continuous genomic structure. Formal stock identification, DAPC, and ADMIXTURE consistently assigned 11 individuals from Portugal and the Canary Islands to the Mediterranean stock. Within the Atlantic, ancestry patterns were more diffuse and included individuals with assignments discordant with their a priori management stock. Heterozygosity was broadly similar among stocks, whereas rarefied allelic richness was lower in the Mediterranean than in either Atlantic stock. Overall, the current management framework captures the major Mediterranean–Atlantic differentiation but only partially reflects the weaker genomic structure within the Atlantic. These findings show that population-genomic analyses can refine stock-structure inference and individual assignment while providing complementary information for swordfish assessment and monitoring.

## 1. Introduction

Effective fisheries management depends on the identification of biologically meaningful population units and on understanding the degree of connectivity among them. This can be particularly challenging in highly migratory marine fishes, where extensive movements, large population sizes and gene flow may generate spatial genetic patterns that are weaker or more complex than the operational boundaries used for stock assessment and management [1,2,3,4,5]. Population-genomic approaches can increase the resolution of these patterns by integrating information on genetic differentiation, ancestry, individual assignment and within-population diversity [3,6].

Swordfish (*Xiphias gladius*) is a large, highly migratory pelagic predator distributed throughout tropical, subtropical and temperate oceans. In the Atlantic Ocean and Mediterranean Sea, the International Commission for the Conservation of Atlantic Tunas (ICCAT) currently manages swordfish as three major stocks: North Atlantic, South Atlantic and Mediterranean [7,8]. The North and South Atlantic management units are separated at 5° N, whereas the Strait of Gibraltar represents the operational boundary between Atlantic and Mediterranean stocks. Given the species’ high dispersal capacity and extensive seasonal movements, defining the extent to which these management units correspond to underlying biological population structure remains particularly relevant for stock assessment and long-term management.

Genetic studies have consistently identified the Mediterranean–Atlantic contrast as the strongest component of swordfish population structure in this region, whereas differentiation within the Atlantic has generally been weaker and less clearly resolved [9,10,11,12,13]. Studies based on mitochondrial DNA, microsatellites and multilocus nuclear markers have supported differentiation between Mediterranean and Atlantic swordfish while also revealing connectivity and admixture within the Atlantic [9,10,11,12,13]. Historical processes have also contributed to the present-day genetic structure of the species. Mitochondrial evidence indicates that Atlantic–Mediterranean differentiation reflects a complex phylogeographic history involving vicariance, secondary contact and demographic expansion associated with Pleistocene climatic oscillations [14]. More recent studies have further documented spatial and temporal variation in genetic diversity within the Mediterranean [12,15]. Together, these studies indicate that swordfish population structure is hierarchical, with a pronounced Mediterranean–Atlantic differentiation superimposed on weaker patterns within the Atlantic.

Despite substantial previous work, the relationship between current ICCAT management units and underlying genomic population structure remains incompletely resolved, particularly within the Atlantic. Broad-scale SNP-based analyses spanning both Atlantic and Mediterranean sampling areas are still limited, and a more extensive genomic framework is needed to evaluate differentiation among management units and individual ancestry patterns across their boundaries. Integrating these analyses with comparisons of genetic diversity can provide a more comprehensive picture of how genetic variation is structured across the species’ range.

Here, we combined a newly assembled swordfish reference genome with ddRAD sequencing of individuals collected across 14 Atlantic and Mediterranean sampling areas. Specifically, we aimed to: (i) characterize genomic population structure and genetic differentiation and assess their correspondence with the current ICCAT management-stock framework; (ii) evaluate individual stock assignment and ancestry patterns across current management boundaries using complementary multivariate, ancestry-based and formal assignment approaches; and (iii) compare genetic-diversity patterns among stocks and sampling areas using standardized population-genetic metrics.

## 2. Results

### 2.1. Sampling Coverage

The initial collection comprised 575 swordfish from 14 sampling areas spanning the North Atlantic, South Atlantic and Mediterranean Sea. Six sampling areas were assigned a priori to ATL-N, three to ATL-S and five to MED according to the current ICCAT management boundaries (Figure 1; Table 1).

### 2.2. Reference-Genome Assembly and Annotation

The final swordfish reference assembly spanned 687.5 Mb and comprised 4493 scaffolds, with an N50 of 9.37 Mb, a maximum scaffold length of 50.25 Mb and an average GC content of 40.7%. BUSCO analysis with the Actinopterygii dataset, used as the primary lineage-specific assessment, identified 93.4% complete orthologs, including 92.1% single-copy and 1.2% duplicated orthologs. Complete BUSCO estimates for the Actinopterygii, Metazoa and Eukaryota datasets are provided in Table S1.

Genome annotation identified 21,660 protein-coding genes and 22,569 high-confidence non-coding genes. Interspersed repeats represented 4.17% of the assembly, including 2.61% DNA transposons and 1.54% retroelements. A total of 148 scaffolds could not be anchored to the chromosome-scale reference and were reported as chromosome 0 for assembly summaries, whereas 535 additional scaffolds remained unassociated with a chromosome (Table S2).

### 2.3. ddRAD Sequencing and Quality Control

ddRAD sequencing generated 719,722,497 paired-end reads. Initial variant and sample filtering retained 519 individuals and 26,324 SNPs. The relatedness and sample-identity assessment subsequently removed 15 duplicate or repeated profiles, producing a final deduplicated dataset of 504 individuals: 261 ATL-N, 103 ATL-S and 140 MED. The available area-level accounting of retained and excluded individuals is reported in Table S3.

Across the 504 retained individuals, median sequencing depth was 11.48× (IQR, 8.01–16.42×), median missing-genotype rate was 0.84% (IQR, 0.26–2.50%) and median SNP call rate was 99.16% (IQR, 97.50–99.74%). Median depth among sampling areas ranged from 7.29× in Rio Grande do Sul to 17.60× in Portugal, whereas median call rate ranged from 95.15% in the Ligurian Sea to 99.69% in Western Sahara. Complete distributions by stock and sampling area are reported in Table S4.

The deduplicated 504-individual × 26,324-SNP panel was retained for diversity analyses. Per-stock HWE filtering, LD pruning and intersection of the three stock-specific locus sets produced a second panel of 504 individuals and 15,049 SNPs for population-structure, genetic-distance, differentiation, ADMIXTURE and assignment analyses. Forty-nine SNPs in this structure panel occurred on unanchored scaffolds (0.33%) and were retained.

### 2.4. Genetic Diversity, Genetic Distance and Differentiation

#### 2.4.1. Genetic Diversity Among Stocks and Sampling Areas

Under the a priori ICCAT management-stock classification, Ho and Hs were broadly comparable among stocks (Table 2). ATL-N showed Ho = 0.229, Hs = 0.239 and FIS = 0.042; ATL-S showed Ho = 0.225, Hs = 0.233 and FIS = 0.034; and MED showed Ho = 0.236, Hs = 0.243 and FIS = 0.029. Rarefied mean allelic richness was lower in MED (1.903) than in ATL-N (1.996) and ATL-S (1.978).

At the sampling-area level, Ho ranged from 0.223 to 0.238, Hs from 0.230 to 0.243 and FIS from 0.019 to 0.042 after exclusion of the 18 focal individuals (Table 3). Rarefied mean allelic richness ranged from 1.603 in Rio Grande do Sul to 1.625 in Western Sahara. The Mediterranean sampling areas showed closely similar mean values (1.608–1.611).

A sensitivity analysis based on genetically informed stock reassignment produced very similar stock-level diversity estimates. Under this classification, ATL-N showed Ho = 0.228, Hs = 0.236 and FIS = 0.035; ATL-S showed Ho = 0.226, Hs = 0.233 and FIS = 0.030; and MED showed Ho = 0.236, Hs = 0.243 and FIS = 0.029. Rarefied mean allelic richness remained lower in MED (1.905) than in ATL-N (1.988) and ATL-S (1.979). At the sampling-area level, inclusion of the focal individuals produced the largest changes in FIS in the Canary Islands and Portugal, while the overall pattern of allelic richness remained unchanged (Table S5).

#### 2.4.2. Genetic Distance and Pairwise Differentiation

Pairwise Prevosti genetic distances were consistent with stronger Mediterranean–Atlantic differentiation than with differentiation between the two Atlantic stocks. The 11 individuals sampled in Portugal and the Canary Islands that were subsequently assigned to MED showed genetic-distance patterns consistent with the Mediterranean group.

Under the a priori ICCAT management-stock classification, pairwise FST was lowest between ATL-N and ATL-S (0.0084) and substantially higher between ATL-N and MED (0.0769) and between ATL-S and MED (0.0996); all three comparisons were significant (*p* < 0.05). A sensitivity analysis based on genetically informed stock reassignment produced very similar estimates (ATL-N vs. ATL-S, 0.0083; ATL-N vs. MED, 0.0830; ATL-S vs. MED, 0.0995).

After excluding the 18 focal individuals, within-stock differentiation among sampling areas was low: pairwise FST ranged from −0.0001 to 0.0014 within ATL-N, from −0.0001 to 0.0018 within ATL-S and from −0.0001 to 0.0013 within MED (Table 4). Comparisons between Mediterranean and Atlantic areas were consistently larger (0.0819–0.1068). The corresponding complete 504-individual matrix based on a priori assignments is provided in Table S6.

### 2.5. Multivariate and Hierarchical Population Structure

#### 2.5.1. Principal Component Analysis and AMOVA

PC1 explained 7.4% of total variance in the unscaled genotype-matrix PCA and 6.9% in the locus-scaled PCA; the corresponding PC2 values were 0.7% and 0.6%. Both analyses separated MED from the two Atlantic stocks along PC1, whereas ATL-N and ATL-S partially overlapped (Figure 2). The area-faceted PCA showed extensive overlap among sampling areas within each stock and no distinct area-level subclusters (Figure S1).

AMOVA confirmed significant stock-level structure: the between-stock component explained 5.85% of total molecular variance (ΦST = 0.059, *p* = 0.001; 999 permutations). The area-within-stock component was significant but small (0.09%; Φ = 0.0010, *p* = 0.001), while the between-individual component within areas was not significant (*p* = 0.136). A sensitivity analysis excluding discordant or ambiguous individuals produced a similar stock-level estimate (ΦST = 0.0624, *p* = 0.001).

#### 2.5.2. Quality-Control Assessment of PC2

The multiple regression of PC2 on technical and biological covariates explained 72% of PC2 variance (adjusted R^2^ = 0.72). Missing-genotype rate (coefficient = 5.85, *p* = 0.22) and mean sequencing depth (coefficient = 0.035, *p* = 0.13) were not significant after accounting for stock. Stock was the dominant predictor (ATL-S versus ATL-N: coefficient = −10.72, *p* = 1.5 × 10^−120^; MED versus ATL-N: coefficient = −3.79, *p* = 3.1 × 10^−33^), while sequencing batch had a smaller residual effect (coefficient = 0.75, *p* = 0.015). Thus, PC2 was not explained by genotype missingness, although a minor batch contribution remained detectable.

#### 2.5.3. Principal Coordinates Analysis

PCoA of the Prevosti-distance matrix explained 11.6% and 0.9% of the variance on coordinates 1 and 2, respectively (Figure 3). The ordination reproduced the PCA pattern, with clear Mediterranean–Atlantic separation and partial overlap between ATL-N and ATL-S, confirming that the principal structure was not specific to a single ordination method.

#### 2.5.4. Discriminant Analysis of Principal Components

The cross-validated DAPC retained 13 principal components and separated the three a priori stocks along two discriminant functions explaining 76.9% and 23.1% of discriminant variance (Figure 4), respectively. Overall reassignment accuracy was 96.4% (486/504): 93.5% for ATL-N, 99.0% for ATL-S and 100% for MED.

Eighteen individuals had a maximum-posterior DAPC assignment different from their a priori stock. These included the 11 ATL-N individuals from the Canary Islands (*n* = 5) and Portugal (*n* = 6) assigned to MED with posterior probabilities >0.999999999; five additional ATL-N individuals from the Equatorial Atlantic (*n* = 3) and Western Sahara (*n* = 2) assigned to ATL-S with posterior probabilities ≥0.9997; one ATL-N individual, SI190678, with a more ambiguous ATL-S posterior probability of 0.73; and one ATL-S individual, ML190057, assigned to ATL-N with posterior probability >0.99. None showed unusually high missingness or low sequencing depth relative to the retained dataset.

### 2.6. ADMIXTURE Analyses

Across the 20 independently seeded global replicates per K, log-likelihoods were essentially invariant for K = 1–3 and became increasingly multimodal at K ≥ 4 (Table S7). Ten-fold cross-validation error decreased sharply from K = 1 (0.4617) to K = 2 (0.42500) and was only marginally lower at K = 3 (0.42426) in the unbalanced dataset. The global ancestry profiles therefore supported a strong primary Mediterranean–Atlantic split at K = 2, with a weaker Atlantic component appearing at K = 3 (Figure 5); the corresponding hierarchical Atlantic-only ancestry profiles are shown in Figure 6.

The sample-size-balanced analysis consistently preferred K = 2 over K = 3 in all ten independent subsamples (Table S8), indicating that the marginal global preference for K = 3 in the raw dataset was sensitive to unequal stock sample sizes. Nevertheless, the Atlantic-only analysis also supported K = 2 within the Atlantic subset (lowest CV error = 0.43139; Table S9), with 96.9% hard-assignment concordance with ATL-N/ATL-S labels. The ancestry coefficients formed a continuum: 31% of individuals were near-pure (Q <0.1 or >0.9), 22% were strongly intermediate (Q = 0.3–0.7) and 6.5% were close to an even 50:50 ancestry profile (Figure 6).

Assignments of the 18 focal individuals were stable across all ten balanced subsamples. The 11 Portugal/Canary Islands individuals retained a mean Mediterranean-associated ancestry coefficient of 0.99999, while the remaining focal individuals remained predominantly Atlantic. These analyses support K = 2 as the primary global structure and a weaker, diffuse ATL-N/ATL-S ancestry gradient as a secondary hierarchical signal.

### 2.7. Genetic Stock Identification

Leave-one-out self-assignment of the 486-individual rubias reference baseline was 100% correct at the stock level. All 18 focal individuals had a maximum posterior stock-assignment probability of 1.00 (Table 5). The 11 individuals from Portugal and the Canary Islands were assigned to MED by rubias, ADMIXTURE and DAPC. Using the final analyzed area sample sizes, these individuals represented 15.8% of Portugal samples (6/38; Wilson 95% CI, 7.4–30.4%) and 12.2% of Canary Islands samples (5/41; Wilson 95% CI, 5.3–25.5%). No individual sampled in the Mediterranean was assigned to an Atlantic stock.

Five ATL-N individuals were assigned to ATL-S across DAPC, hierarchical ADMIXTURE and rubias, while ML190057 showed the opposite ATL-S-to-ATL-N assignment. SI190678 was assigned to ATL-S by rubias but showed intermediate Atlantic ancestry in ADMIXTURE (ATL-S Q = 0.59) and a less decisive DAPC posterior probability (0.73), and was therefore treated as an ambiguous Atlantic individual rather than a confident migrant. CT193310 was consistently assigned to ATL-S, but its low rubias z-score percentile indicated a comparatively poor fit to that reference stock. Bootstrap uncertainty for the primary Mediterranean–Atlantic distinction was small (SE ≤ 0.011).

Rubias convergence diagnostics supported the stability of these assignments: effective sample sizes ranged from 1827 to 2244, Geweke z-scores ranged from −1.81 to 1.81 and the three independently seeded chains agreed within 0.005 in estimated mixing proportions. Increasing the analysis to 50,000 iterations with a 10,000-iteration burn-in did not change the top assignment of any focal individual (Table S10).

## 3. Discussion

Previous genetic studies established that Atlantic and Mediterranean swordfish are not genetically homogeneous and suggested that their biological distributions do not fully coincide with current management boundaries [9,10,11,12,13,14,16]. The present study advances this framework by resolving, with thousands of nuclear SNPs, the markedly unequal strength of differentiation among the three ICCAT stocks. The dominant genomic discontinuity separates Mediterranean from Atlantic swordfish, whereas the North–South Atlantic signal is much weaker and is expressed as a continuous ancestry transition rather than as a sharp population break. This distinction, together with formal individual stock assignment across management boundaries, represents the main contribution of the present study. It shifts the interpretation from a simple three-stock model toward a hierarchical view in which current management units correspond to biologically different levels of population differentiation.

The Mediterranean–Atlantic contrast is the most robust component of swordfish population structure and has been repeatedly recovered using different classes of molecular markers. Early mitochondrial analyses identified strong heterogeneity among Mediterranean, North Atlantic and South Atlantic swordfish and revealed deeply divergent maternal lineages [14,16]. Subsequent microsatellite studies confirmed substantial differentiation between Mediterranean and Atlantic samples [10,13], while Smith et al. [9] used 26 SNPs in 10 nuclear genes to distinguish the three ICCAT stocks and to investigate connectivity and admixture across their boundaries. Righi et al. [13] later reported an Atlantic–Mediterranean \(F_{ST}\) of approximately 0.09 using microsatellites, similar in magnitude to the differentiation observed in the present study. The present study extends this earlier evidence by showing, across a broader geographic sampling design and thousands of nuclear SNPs, that Mediterranean–Atlantic differentiation remains the dominant component of population structure.

The historical depth of this differentiation is also important for interpreting the present genomic pattern. Bremer et al. [14] showed that the Atlantic–Mediterranean phylogeography of swordfish reflects a complex combination of vicariance, population expansion, secondary contact and introgression rather than a simple contemporary separation between basins. This historical framework helps explain why strong population differentiation can persist despite the considerable dispersal capacity of swordfish. Electronic tagging studies have documented large seasonal movements and connectivity among Atlantic feeding and spawning areas [17,18,19]. Abascal et al. [17] recorded movements linking central, north-western, south-eastern and north-eastern Atlantic regions, including trajectories exceeding several thousand kilometers, while Braun et al. [19] showed complex connectivity patterns across the North Atlantic using satellite tags, conventional tagging and fisheries data. Extensive movement therefore does not necessarily imply equivalent reproductive gene flow. Spatial or temporal structuring of spawning can maintain genetic differentiation even when individuals overlap during feeding or migration, a distinction increasingly recognized in marine population genomics [3,4,5].

The main refinement provided by the present study becomes particularly evident within the Atlantic. Smith et al. [9] had already shown significant differentiation between North and South Atlantic swordfish and argued that the 5° N management boundary did not fully capture the inferred genetic distribution of the two populations. Their analysis, however, was based on a relatively small multilocus panel of 26 SNPs. Our reduced-representation population-genomic dataset resolves this signal differently. The North–South Atlantic contrast is detectable, but substantially weaker than the Mediterranean–Atlantic division and characterized by extensive overlap and intermediate ancestry. This suggests that ATL-N and ATL-S should not be interpreted as genomic equivalents of the Mediterranean and Atlantic components. Instead, the Atlantic pattern is more consistent with weak spatial structure superimposed on substantial connectivity.

This distinction is biologically important because low but significant differentiation in highly abundant marine fishes does not necessarily indicate sharply isolated populations. Large effective population sizes, high dispersal and recurrent gene flow can maintain low \(F_{ST}\) even when some spatial structure exists [4,5]. The Atlantic ancestry continuum observed here could arise through recurrent gene flow, isolation by distance, historical connectivity, or a combination of these processes. Formally distinguishing among these spatial and demographic processes will require dedicated analyses incorporating geographic distance and broader spatial sampling and could be addressed in future work together with a more detailed assessment of spatial patterns in genetic diversity. In that context, the biological issue associated with the 5° N boundary is not simply whether the line should be shifted northward, southward or diagonally. A fixed management delimiter may necessarily simplify a population transition that is intrinsically continuous. This interpretation also fits the movement literature: Neilson et al. [18] found strong seasonal movements within the western North Atlantic but little evidence of basin-wide mixing, whereas Braun et al. [19] identified limited connectivity among several North Atlantic regions and noted discrepancies between movement patterns and existing stock assumptions.

The comparison with recent genomic work on swordfish in the Indian Ocean further illustrates the value of high-density SNP data. Chevrier et al. [20] analyzed 1694 swordfish from 24 locations and detected two broad genomic components in the Indian Ocean, despite the species having been treated there under a simpler management framework. Their results showed that large SNP datasets can reveal population structure that was difficult to resolve with earlier markers and, importantly, can distinguish broad neutral structure from stronger signals associated with loci potentially under selection. Although the Atlantic–Mediterranean and Indian Ocean systems have different demographic histories, both studies demonstrate that high dispersal in swordfish does not preclude spatially structured genomic variation. The contribution of the present study is to apply that population-genomic resolution specifically to the three Atlantic–Mediterranean ICCAT stocks and to show that their genomic distinctiveness is strongly asymmetric.

A second major advance is the transition from population-level differentiation to explicit individual stock assignment. Previous swordfish studies had already detected admixture and suggested that Mediterranean-affiliated individuals could occur west of Gibraltar [9,13]. Building on previous evidence of Atlantic–Mediterranean overlap [9,13], the present study provides higher-resolution individual assignments supported concordantly by rubias, DAPC and ADMIXTURE. Eleven swordfish sampled in Portugal and the Canary Islands were consistently assigned to the Mediterranean reference stock by rubias, DAPC and ADMIXTURE, and the assignment baseline showed complete leave-one-out classification at the stock level. This makes it possible to move from a qualitative observation of admixture toward a genomic stock-identification framework in which individual affiliation and uncertainty can be quantified.

This distinction between population structure and individual stock identification is increasingly central to fisheries genomics. Population structure establishes whether biological populations differ; genetic stock identification determines the most likely biological origin of individuals captured in potentially mixed fisheries. Andersson et al. [3] emphasized that one of the principal applications of population genomics in fisheries is precisely the development of validated genetic baselines that can subsequently be used to estimate stock composition in mixed samples. This approach has already moved from research to operational application in several North Atlantic fisheries. In Atlantic herring, genome-derived SNP panels have been used to distinguish biological populations that overlap outside spawning areas and to quantify their contribution to mixed catches [21]. Seljestad et al. [22], analyzing more than 15,000 herring, subsequently demonstrated that genetically defined populations may occur well outside their nominal management areas and that static geographic boundaries can therefore poorly represent the composition of migratory aggregations. Similarly, year-round genetic monitoring of Atlantic cod in West Greenland has allowed stock-specific contributions to mixed fisheries to be quantified and linked directly to management decisions [23].

The 11 Mediterranean-assigned swordfish detected in Portugal and the Canary Islands should be interpreted in this framework. Their occurrence does not undermine the strong Mediterranean–Atlantic differentiation. Rather, it demonstrates that geographic stock boundaries and the distribution of individuals belonging to those populations are not identical. Strong population differentiation can coexist with spatial overlap outside reproductive areas. The management significance therefore depends less on the presence of individual discordant assignments than on whether such occurrences are temporally persistent and represent a non-negligible component of particular fisheries. Repeated genomic sampling in Portugal, the Canary Islands and adjacent eastern Atlantic waters would allow this distinction to be tested. If Mediterranean-associated fish occur consistently across seasons or years, genomic stock identification could eventually quantify their contribution to catches; if their occurrence is highly episodic, the management implications would be more limited.

The Atlantic discordant assignments have a different biological meaning because the underlying ATL-N/ATL-S differentiation is weak. Several individuals were consistently associated with the opposite Atlantic reference group, but these assignments occur against a background of continuous ancestry. High statistical assignment probability should therefore not be equated with strong biological isolation of the reference groups. In this case, the assignments reinforce the interpretation of a permeable Atlantic transition rather than demonstrating discrete movements between two deeply differentiated populations. This difference between the Mediterranean–Atlantic and North–South Atlantic assignment contexts is itself an important outcome of the denser genomic dataset and was much more difficult to evaluate with the smaller marker panels used previously.

The diversity analyses provide a third, complementary contribution. Mediterranean swordfish showed lower rarefied allelic richness than either Atlantic stock, whereas heterozygosity was broadly comparable. Earlier studies had already reported lower genetic diversity in Mediterranean swordfish using both mitochondrial and microsatellite markers. Righi et al. [13] found lower allelic richness and expected heterozygosity in Mediterranean samples relative to Atlantic swordfish, while Righi et al. [12] subsequently reported temporal reductions in mitochondrial haplotype diversity and female relative effective population size. The present nuclear SNP results therefore extend previous evidence that Mediterranean swordfish differ in standing genetic diversity from Atlantic populations.

The important advance here is methodological and interpretative. The current comparison is based on thousands of nuclear SNPs and standardized rarefaction, reducing the influence of unequal sample sizes on allelic-richness estimates. However, the observed diversity pattern alone does not identify its underlying demographic cause. Allelic richness and heterozygosity capture different components of neutral genetic variation, with allelic richness being particularly sensitive to low-frequency variants [24,25]. Contemporary differences may therefore reflect long-term effective population size, historical isolation, population subdivision, migration, recent demographic change, or combinations of these processes. The historical Atlantic–Mediterranean divergence documented by Bremer et al. [14] makes such demographic alternatives particularly relevant.

Distinguishing among these explanations will require explicit demographic inference. Site-frequency-spectrum or coalescent-based approaches could compare alternative histories of divergence, gene flow and effective population-size change and determine whether the lower Mediterranean allelic richness can be explained primarily by long-term demographic history or whether more recent contraction is required. Such analyses would allow the relative contributions of historical and more recent demographic processes to the observed diversity pattern to be evaluated explicitly.

The reduced-representation nature of ddRAD sequencing defines the scale of inference of the present study. The present dataset samples thousands of nuclear loci distributed across the reference genome and provides substantially greater resolution than previous Atlantic–Mediterranean swordfish studies based on tens of loci, but it does not constitute uniform whole-genome coverage. Accordingly, its principal strength lies in resolving population structure, individual assignment and stock-level diversity rather than exhaustive locus-specific or functional variation. This distinction is consistent with current fisheries-genomics frameworks, which emphasize that reduced-representation genomic datasets can be highly effective for defining baselines and identifying mixed stocks even when they do not provide complete whole-genome representation [3].

Taken together, the present results provide a higher-resolution interpretation of the existing understanding of swordfish population structure.

Earlier mitochondrial, microsatellite and multilocus studies correctly identified strong Mediterranean–Atlantic differentiation, weaker differentiation within the Atlantic and some degree of spatial mixing [9,10,11,12,13,14,16]. What the present SNP dataset adds is a more resolved hierarchical interpretation of these patterns. The Mediterranean–Atlantic contrast represents a strong and historically established genomic division that can coexist with spatial overlap of individuals, whereas the North–South Atlantic contrast is a much weaker and more continuous form of structure. Formal individual assignment further shows that these two situations cannot be interpreted in the same way.

This distinction has practical implications for fisheries management. The results support the Mediterranean–Atlantic division as the principal biological separation represented by the current ICCAT framework, while indicating that the North–South Atlantic boundary corresponds to a substantially weaker genomic transition. At present, the most direct management application of these results is therefore to complement geographic stock designation with genomic monitoring in areas of potential stock overlap. Portugal and the Canary Islands are particularly suitable for temporally replicated stock-identification surveys, while denser spatial sampling across the eastern tropical Atlantic could clarify the stability and extent of the North–South ancestry gradient. In other exploited fishes, such genetic baselines have become management tools only after repeated validation of mixed-stock composition across years and fisheries [21,22,23]. Recent syntheses similarly emphasize that genomics contributes most effectively to fisheries policy when it addresses specific operational questions—such as stock composition, stock-specific exploitation or monitoring of biological units—rather than being treated as a stand-alone indicator [3].

Overall, this study provides a higher-resolution characterization of the biological relationships underlying the current Atlantic–Mediterranean swordfish management framework. By moving from small multilocus panels to thousands of nuclear SNPs, the study demonstrates that the three ICCAT stocks are not genomically equivalent, resolves the weak and continuous nature of North–South Atlantic structure, provides a formal framework for individual stock assignment across management boundaries, and establishes a standardized nuclear-genetic diversity baseline. These advances provide a more realistic representation of swordfish population structure and create a foundation for integrating genomic stock identification with tagging, reproductive biology and conventional stock assessment.

## 4. Materials and Methods

### 4.1. Sampling Activities

A total of 575 swordfish (Xiphias gladius) from 14 sampling areas in the Atlantic Ocean and Mediterranean Sea were included in the initial dataset (Figure 1; Table 1). The ICCAT management boundaries at 5° N, separating the North and South Atlantic stocks, and at the Strait of Gibraltar, separating the North Atlantic and Mediterranean stocks, were used for the a priori assignment of sampling localities to three management stocks: North Atlantic (ATL-N), South Atlantic (ATL-S), and Mediterranean (MED). Fish were collected between 2016 and 2020 by commercial fishing vessels or research vessels using longlines, within the ICCAT Swordfish Year Programme project “Biological samples collection for growth, reproduction and genetic studies”.

For each specimen, a piece of caudal or anal fin or skeletal muscle was collected, preserved in absolute ethanol, and stored at −20 °C until DNA extraction for ddRAD sequencing. A 1.5 mL blood sample was collected from one swordfish caught in the Adriatic Sea and stored at −20 °C until DNA extraction for reference-genome sequencing.

### 4.2. Reference-Genome Generation and Annotation

A chromosome-anchored reference assembly was generated from the Adriatic Sea individual using a hybrid sequencing strategy combining Oxford Nanopore MinION (Oxford, UK) long reads (approximately 20× coverage) and Illumina NovaSeq 6000 (San Diego, CA, USA) paired-end reads (approximately 30× coverage). Read-level contamination screening was performed with Centrifuge v1.0.4-beta before genome assembly; full details of the reference database and filtering procedure are provided in Supplementary Methods S1. Long-read assemblies generated independently with Canu [26] and wtdbg2 [27] were corrected with Illumina reads, merged, and subjected to successive short-read and RNA-seq-guided polishing steps. Assembly quality was evaluated with QUAST [28], and the polished scaffolds were anchored to the previously published chromosome-scale swordfish assembly [29] using RaGOO [30]. Structural and functional annotation integrated repeat masking, published swordfish RNA-seq evidence [31], ab initio gene prediction, protein homology, and functional annotation against Gene Ontology and KEGG resources. Genome completeness was evaluated with BUSCO [32]. Complete software settings and parameters are provided in Supplementary Methods S1.

### 4.3. ddRAD Sequencing, Read Processing, and Variant Calling

Genomic DNA was extracted from approximately 25 mg of fin or muscle tissue using the Quick-DNA Miniprep Kit (Zymo Research, Irvine, CA, USA), following the manufacturer’s protocol. DNA purity and concentration were assessed with a NanoPhotometer P330 (Implen, Munich, Germany). Double-digest restriction-site-associated DNA libraries were prepared by IGA Technology Services (Udine, Italy) using SphI and MboI and were sequenced on an Illumina NovaSeq 6000 platform. A total of 719,722,497 paired-end reads were generated.

Raw reads were processed with BBDuk [33] to remove adapters and low-quality bases, using a minimum Phred score of 30 and a minimum retained read length of 35 bp. Cleaned reads were aligned to the swordfish reference genome with minimap2 [34]. BAM files were sorted by genomic coordinates, PCR duplicates were flagged with SAMBAMBA [35], and read-group information was added with Picard.

Variants were called with Platypus [36], requiring at least four supporting reads, mapping quality (MAPQ) > 10, and base quality (BQ) > 30; only variants marked PASS were retained. No additional per-genotype filtering based on genotype quality (GQ) was applied after variant calling because sequencing coverage was relatively moderate. VCFtools (v0.1.17) [37] was then used to retain loci with a per-site missing-genotype rate ≤10%, mean site depth ≥4×, and minor-allele frequency ≥0.05. Individuals with more than 10% missing genotypes were excluded. These filters yielded an intermediate dataset of 519 individuals and 26,324 SNPs.

### 4.4. Sample Identity, Relatedness Quality Control, and Analytical Datasets

Pairwise KING-robust kinship coefficients (φ) were calculated for the 519 retained individuals with VCFtools [37]—relatedness2. Pairs with φ > 0.40, consistent with duplicate samples or repeated sequencing of the same individual, were grouped into duplicate clusters. Within each cluster, the individual with the lowest per-sample missing-genotype rate, calculated with VCFtools—missing-indv, was retained. This procedure removed 15 individuals and produced a deduplicated dataset of 504 individuals: 261 ATL-N, 103 ATL-S, and 140 MED.

For the 504 retained individuals, sequencing depth, genotype missingness, and SNP call rate, calculated as 1 minus the missing-genotype rate, were summarized overall and by management stock and sampling area. Distributions were described using medians and interquartile ranges and are reported in Table S4.

Two datasets were used for downstream analyses. Genetic-diversity analyses used the deduplicated 504-individual, 26,324-SNP dataset without HWE filtering or LD pruning. This dataset was retained because removing loci on the basis of within-stock HWE would directly affect FIS estimates. Population-structure, genetic-distance, differentiation, ADMIXTURE, and assignment analyses used a conservative HWE- and LD-filtered panel. For this panel, the 504 individuals were divided according to their a priori stock assignment. Within each stock, SNPs departing from HWE at *p* < 0.05 were removed with VCFtools [37], and LD pruning was performed with bcftools [38] +prune using r^2^ < 0.2 in 50 kb sliding windows. The three stock-specific sets were intersected with bcftools [38] isec (−*n* = 3), and genotypes at the shared positions were extracted from the complete 504-individual VCF. The resulting population-structure dataset comprised 504 individuals and 15,049 SNPs. Thus, the 26,324-SNP diversity dataset was not LD-pruned.

Unanchored scaffolds were retained as separate scaffolds rather than concatenated into an artificial chromosome 0 during population-genetic filtering. Forty-nine of the 15,049 SNPs in the final structure dataset (0.33%) occurred on unanchored scaffolds and were retained in all population-genetic analyses reported here.

### 4.5. Genetic Diversity, Genetic Distance, and Differentiation

Observed heterozygosity (Ho), within-population gene diversity (Hs), and the inbreeding coefficient (FIS) were calculated with hierfstat [39] from the deduplicated, non-HWE-filtered 504-individual, 26,324-SNP dataset. Statistics were estimated separately for the three a priori management stocks and the 14 sampling areas.

Allelic richness was calculated with PopGenReport [40] from the same 504-individual, 26,324-SNP dataset. To account for unequal sample sizes, allelic-richness estimates were standardized by rarefaction. Stock-level estimates were rarefied to 103 individuals, corresponding to the size of the smallest stock sample (ATL-S), whereas sampling-area estimates were rarefied independently to 10 individuals, corresponding to the smallest sampling-area sample (Rio Grande do Sul). The resulting rarefied estimates are reported in Table 2 and Table 3.

Pairwise Prevosti genetic distances among the 504 individuals were calculated from the 15,049-SNP population-structure panel. Pairwise differentiation among management stocks and among sampling areas was estimated with the Weir and Cockerham FST estimator in StAMPP v1.5 [41] using the same SNP panel. Statistical support was assessed with 100 locus-bootstrap replicates, with *p* < 0.05 considered significant.

### 4.6. Multivariate and Hierarchical Population-Structure Analyses

Principal component analysis (PCA) was performed directly on the genotype-dosage matrix, coded as 0, 1, or 2 minor alleles per individual and locus, using the 504-individual, 15,049-SNP panel. Residual missing genotype values were imputed by per-locus mean imputation, with each missing value replaced by the mean allele dosage calculated for that locus across all individuals. Two complementary analyses were calculated by singular-value decomposition of the mean-centered dosage matrix: an unscaled PCA and a locus-scaled PCA following the eigenanalysis framework of Patterson et al. [42], in which each locus was divided by its expected standard deviation under HWE. The analyses were implemented with adegenet (v. 2.1.11) [43].

Principal coordinates analysis (PCoA) was performed with ape::pcoa() [44] on the Prevosti genetic-distance matrix calculated from the same 504 individuals and 15,049 SNPs. No correction for negative eigenvalues was applied because none were present. To determine whether PC2 reflected technical variation, individual PC2 scores were analyzed by multiple linear regression using missing-genotype rate, mean sequencing depth, sequencing batch, and a priori stock as predictors (PC2~missingness + depth + batch + stock).

Discriminant analysis of principal components (DAPC) was performed with adegenet [43] on the same mean-imputed, unscaled genotype-dosage matrix using the three a priori stocks as predefined groups. The number of retained principal components was selected by α-score optimization and verified by stratified 90%/10% cross-validation, with five replicates for each candidate value n.pca = 5, 10, 13, and 20. The final DAPC retained 13 principal components and two discriminant functions.

Analysis of molecular variance (AMOVA) was performed with poppr::poppr.amova() [45] using method = ‘ade4’. Sampling area was nested within stock, and the within-individual variance component was included. Statistical significance was evaluated with 999 permutations. The analysis was applied to the complete 504-individual structure dataset and repeated after excluding individuals with discordant or ambiguous multivariate assignments.

### 4.7. ADMIXTURE Analyses

Population structure and individual ancestry were evaluated with ADMIXTURE v1.4.0 [46]. All ADMIXTURE analyses used the 504-individual, 15,049-SNP population-structure panel. In the global analysis, K = 1–6 was evaluated with 20 independently seeded replicates per K without cross-validation to assess log-likelihood reproducibility, plus five additional replicates per K with 10-fold cross-validation (-cv = 10), for a total of 150 runs.

Atlantic differentiation was subsequently examined using a hierarchical Atlantic-only analysis. The subset comprised 353 individuals (250 ATL-N and 103 ATL-S), excluding the 11 Mediterranean-genotype individuals sampled in North Atlantic areas but retaining individuals reassigned between the two Atlantic stocks. Values of K = 1–4 were tested using the same design of 20 independently seeded replicates and five additional 10-fold cross-validation replicates per K, for a total of 100 runs.

Sensitivity to unequal stock sample sizes was assessed using ten independently generated balanced datasets. After exclusion of the 18 focal or ambiguous individuals, the reference pool contained 140 MED, 244 ATL-N, and 102 ATL-S individuals. Each balanced dataset contained 102 randomly sampled MED individuals, 102 randomly sampled ATL-N individuals, all 102 ATL-S individuals, and all 18 focal individuals (*n* = 324). Distinct random seeds were used for the ten subsamples. Within each subsample, K = 2 and K = 3 were analyzed with five replicates without cross-validation, and K = 1–3 was analyzed with two 10-fold cross-validation replicates, giving 160 runs in total.

Assignment uncertainty was quantified on the complete dataset at K = 2 and K = 3 using ADMIXTURE’s bootstrap option with 200 SNP-resampling replicates (−B 200), producing a standard error and bias estimate for each individual ancestry coefficient. Q matrices from the 20 independent replicates were aligned by testing component permutations and selecting the alignment that maximized cross-replicate correlation. Aligned ancestry coefficients were plotted with pophelper::plotQ [47]. Fixed and recorded random seeds were used throughout.

### 4.8. Genetic Stock Identification

Formal genetic stock identification was performed with rubias v0.4.1 [48] in R 4.3.3 [49]. The reference baseline comprised 486 individuals after excluding 18 focal or ambiguous individuals from the 504-individual panel; management stock was defined as the reporting unit and the 14 sampling areas as collections. Baseline resolving power was evaluated by leave-one-out self-assignment with self_assign().

The 18 excluded individuals were then treated as an unknown mixture. These comprised the 11 Mediterranean-genotype individuals previously identified in Portugal and the Canary Islands, five additional individuals flagged by DAPC in the Equatorial Atlantic and Western Sahara, one DAPC-ambiguous individual (SI190678), and one individual showing an opposite Atlantic reassignment (ML190057). Individual assignments were estimated with infer_mixture() using three independent MCMC chains with distinct seeds, 2000 iterations, and a burn-in of 200 iterations. Assignment goodness of fit was evaluated using the Anderson z-score calibrated against the leave-one-out likelihood distribution of the 486 reference individuals belonging to their assigned stock.

Convergence was evaluated on post-burn-in mixing-proportion traces using effective sample size (coda::effectiveSize) [50], Geweke diagnostics (coda::geweke.diag), and agreement among the three independent chains. An extended sensitivity analysis was also performed with 50,000 iterations, a burn-in of 10,000 iterations, and three new independently seeded chains, and was compared with the initial analysis. PLINK 2 [51] was used for genotype subsetting and format conversion; additional data processing used coda, dplyr, and tidyr. All random seeds were fixed and logged.

For Portugal and the Canary Islands, the observed frequency of Mediterranean-genotype individuals was calculated as the number identified divided by the total number of genotyped individuals from the corresponding sampling area. Two-sided 95% confidence intervals were calculated using the Wilson score method for a binomial proportion.

## 5. Conclusions

This study refines the biological interpretation of the three ICCAT swordfish management stocks by showing that they do not represent equivalent levels of genomic differentiation. The strongest and most consistent division separates Mediterranean from Atlantic swordfish, whereas North–South Atlantic structure is substantially weaker and is better described as a continuous ancestry transition than as a sharp genomic discontinuity. The high-density SNP dataset therefore extends previous swordfish genetic studies by resolving the hierarchical nature of population structure across the Atlantic–Mediterranean system and by providing a formal framework for individual stock assignment.

Individual assignment further showed that capture location does not always coincide with genomic stock affiliation, as demonstrated by Mediterranean-assigned individuals sampled in Portugal and the Canary Islands. In contrast, discordant assignments within the Atlantic occur against a background of weak differentiation and continuous ancestry and should therefore be interpreted as evidence of permeability across the current North–South framework rather than as discrete exchange between strongly separated populations. Genetic-diversity analyses also identified lower rarefied allelic richness in the Mediterranean stock despite broadly similar heterozygosity, providing a standardized baseline for future analyses of the demographic processes underlying this pattern.

Overall, the current ICCAT framework captures the major Mediterranean–Atlantic biological division but only partially represents the weaker genomic structure within the Atlantic. The immediate value of genomic information lies in complementing geographic stock designation through repeated genetic stock identification in areas of potential overlap, particularly the North-East Atlantic. Integration of genomic assignment with temporal sampling, tagging, reproductive information and conventional stock-assessment data could provide a more accurate representation of swordfish population connectivity and mixed-stock composition and strengthen future monitoring of this highly migratory species.

## Figures and Tables

**Figure 1:**
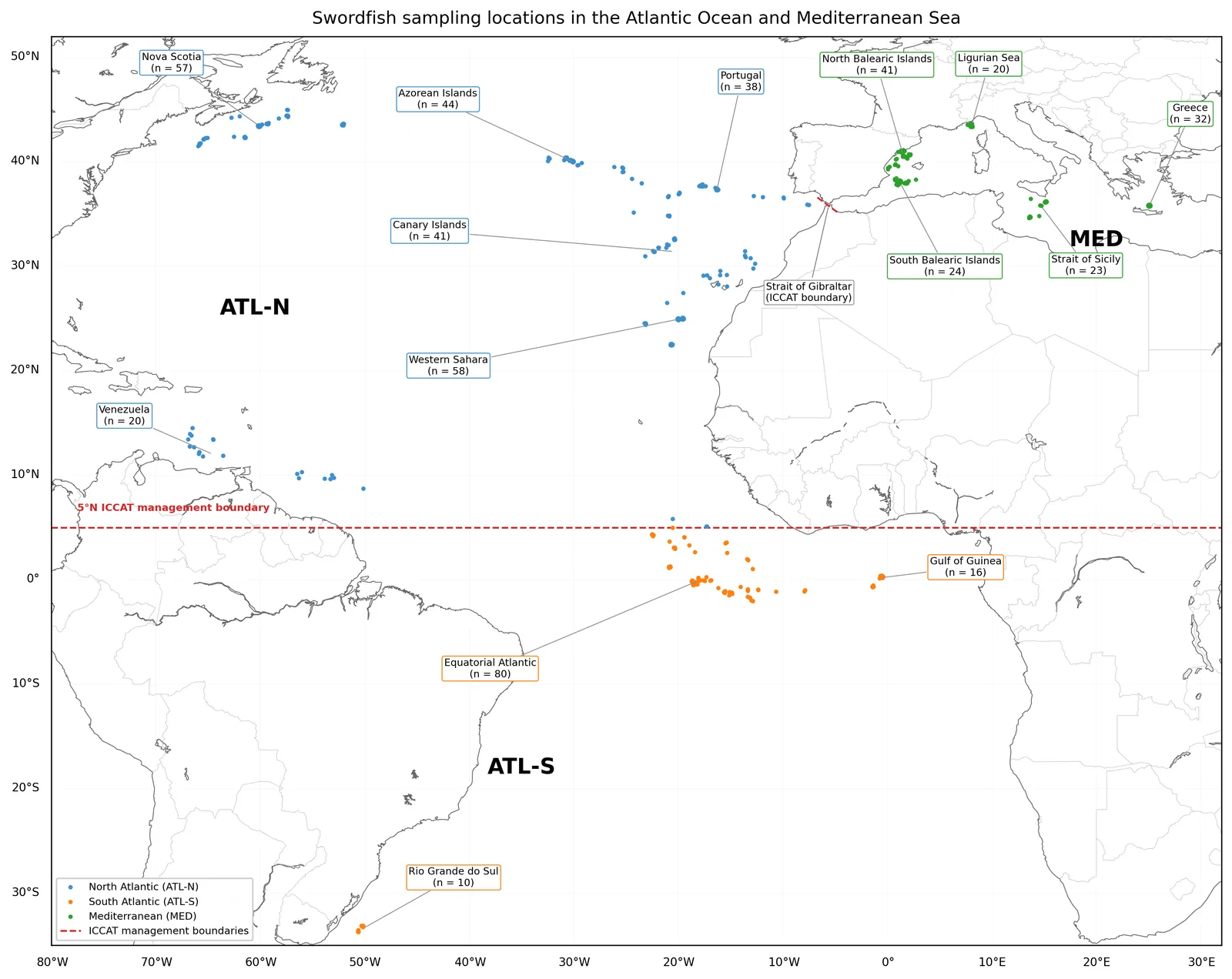
Geographic distribution of the 14 swordfish sampling areas in the Atlantic Ocean and Mediterranean Sea. Colors indicate the three a priori ICCAT management stocks (ATL-N, ATL-S and MED). Dashed red lines indicate the current ICCAT management boundaries at 5° N and at the Strait of Gibraltar.

**Figure 2:**
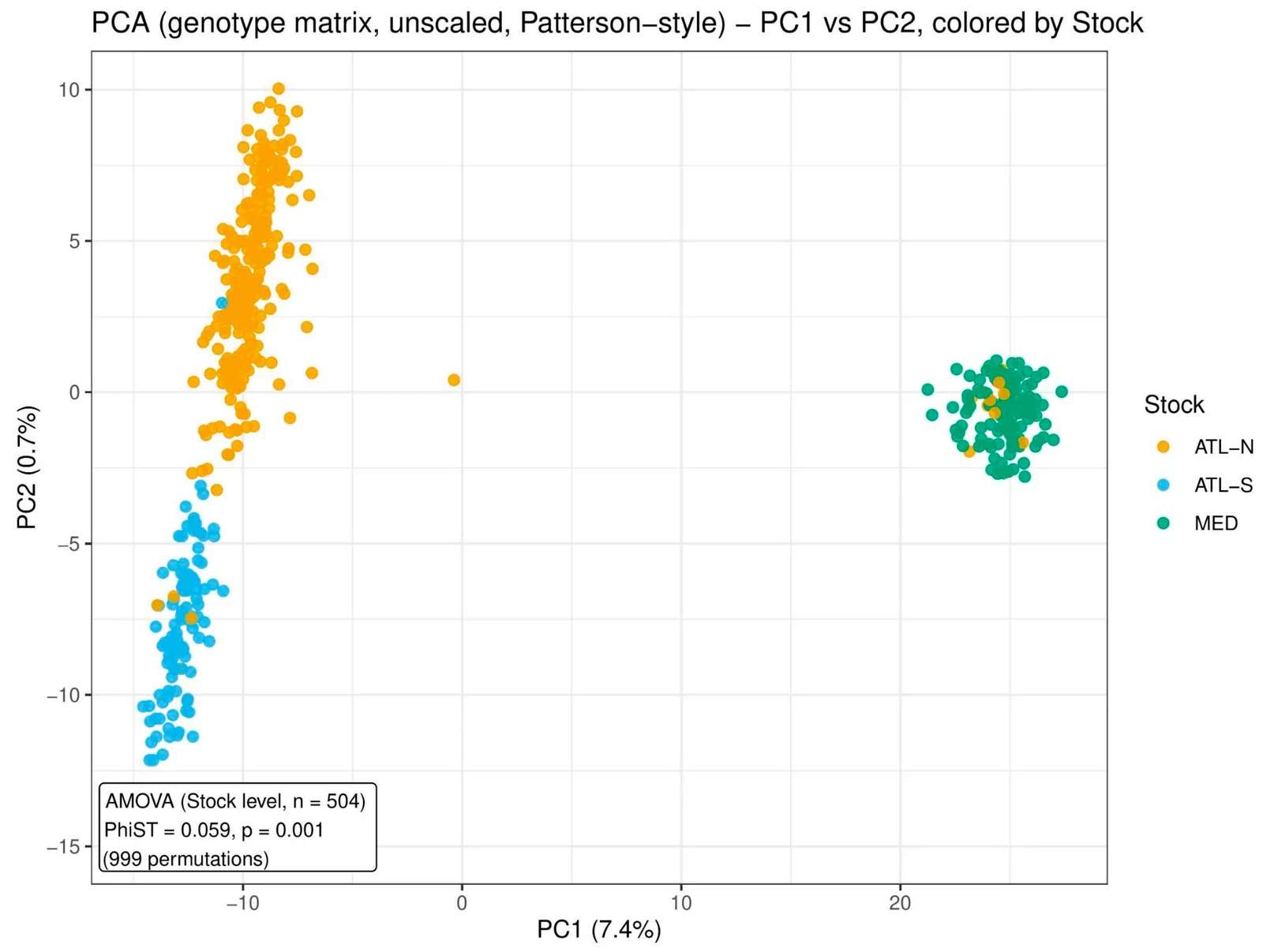
Principal component analysis (PCA) of 504 swordfish based on the LD-pruned 15,049-SNP dataset, colored according to the three a priori ICCAT management stocks (ATL-N, ATL-S and MED). PC1 and PC2 explain 7.4% and 0.7% of total genetic variance, respectively. The inset summarizes the stock-level AMOVA results.

**Figure 3:**
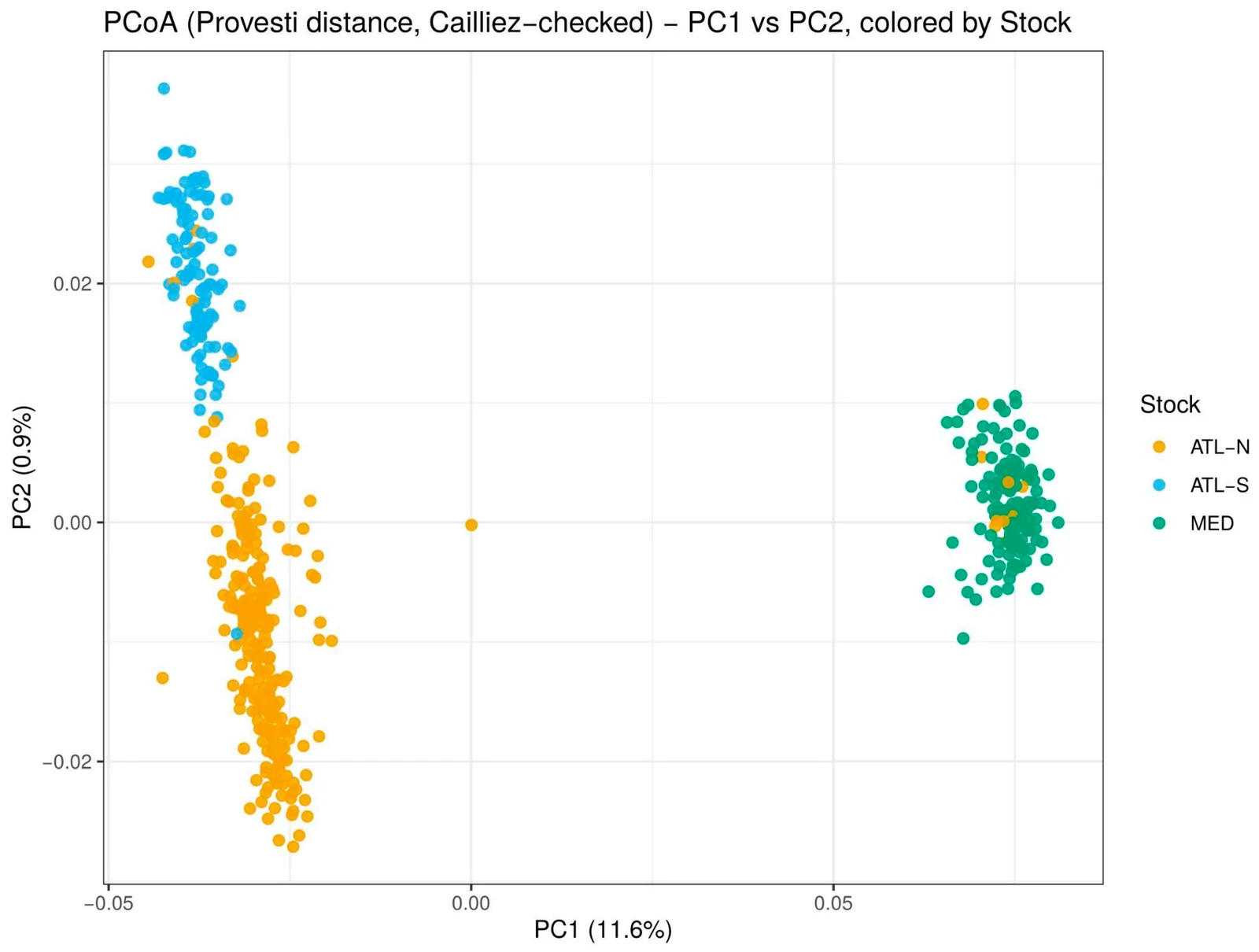
Principal coordinates analysis (PCoA) of the Prevosti genetic-distance matrix for 504 swordfish based on the LD-pruned 15,049-SNP dataset. Individuals are colored according to the three a priori ICCAT management stocks (ATL-N, ATL-S and MED). Coordinate 1 and Coordinate 2 explain 11.6% and 0.9% of the total variation, respectively.

**Figure 4:**
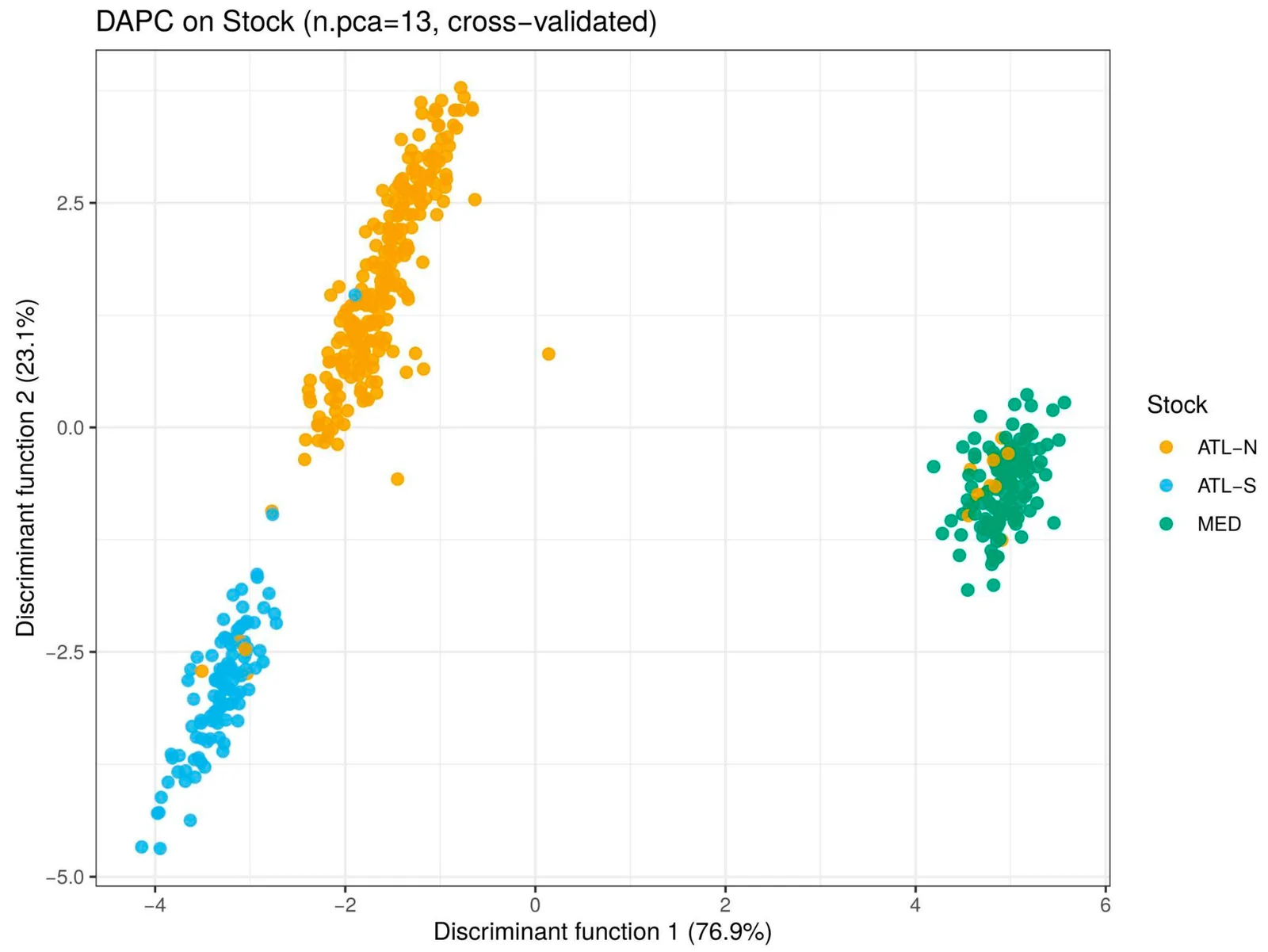
Discriminant analysis of principal components (DAPC) of 504 swordfish based on the LD-pruned 15,049-SNP dataset, using the three a priori ICCAT management stocks (ATL-N, ATL-S and MED) as predefined groups. Thirteen principal components were retained following α-score optimization and cross-validation. The first and second discriminant functions explain 76.9% and 23.1% of the discriminant variance, respectively.

**Figure 5:**
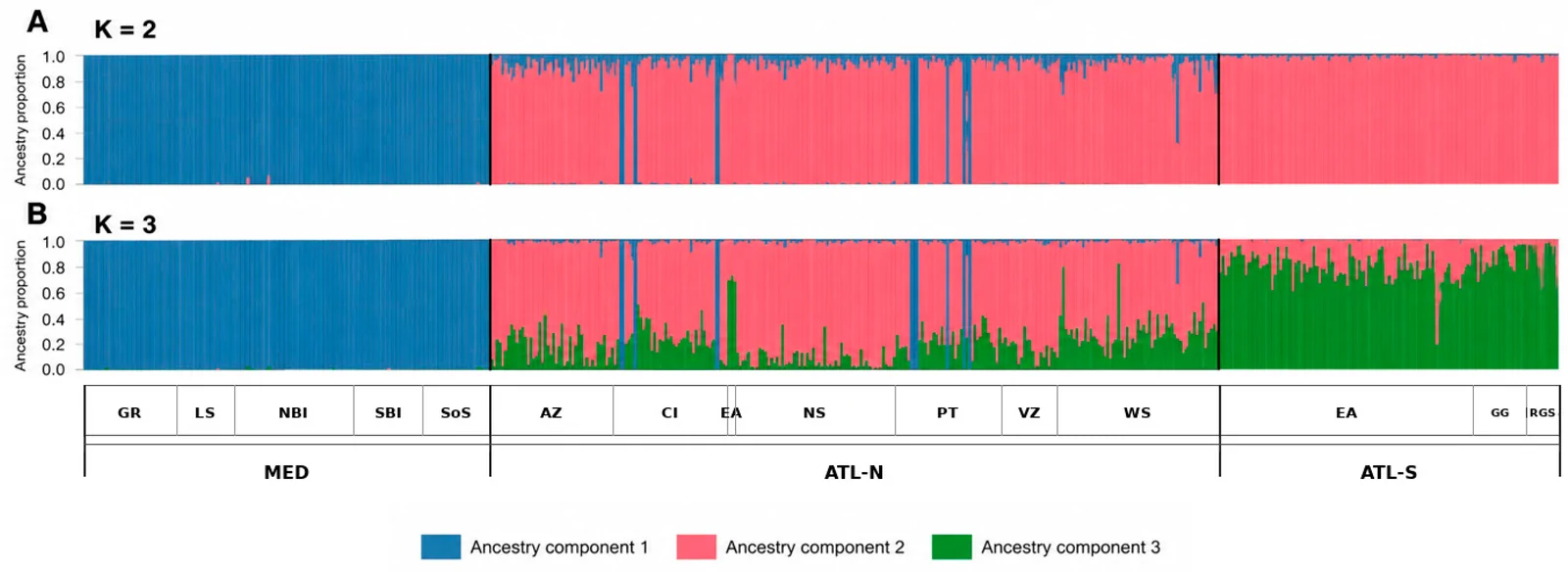
Global ADMIXTURE ancestry profiles for 504 swordfish based on the LD-pruned 15,049-SNP dataset at K = 2 (**A**) and K = 3 (**B**). Individuals are ordered according to their a priori ICCAT management stock and sampling area. Sampling-area abbreviations are shown on the first level below the plots, while the corresponding a priori management stocks are shown on the second level. Black vertical lines indicate the boundaries between ATL-N, ATL-S and MED. Q matrices were aligned across 20 independent replicates for each K. Sampling-area abbreviations: NS, Nova Scotia; AZ, Azorean Islands; WS, Western Sahara; CI, Canary Islands; PT, Portugal; VZ, Venezuela; GG, Gulf of Guinea; EA, Equatorial Atlantic; RGS, Rio Grande do Sul; NBI, North Balearic Islands; SBI, South Balearic Islands; LS, Ligurian Sea; SoS, Strait of Sicily; GR, Greece.

**Figure 6:**
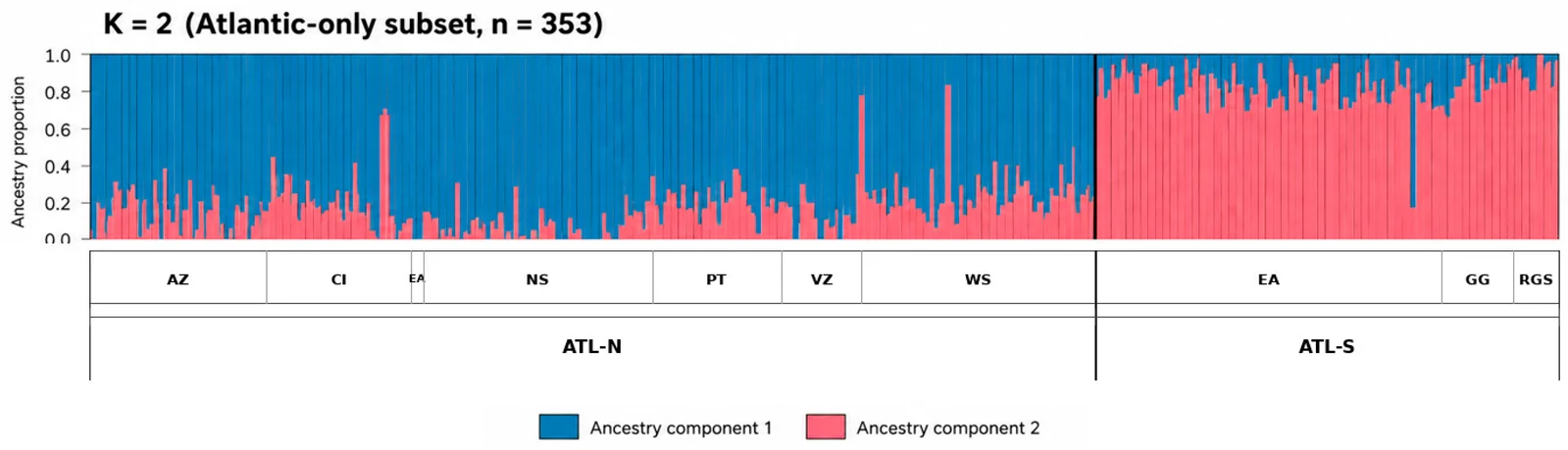
Hierarchical Atlantic-only ADMIXTURE analysis at K = 2 based on 353 swordfish (250 ATL-N and 103 ATL-S) from the LD-pruned 15,049-SNP dataset, after exclusion of the 11 Mediterranean-genotype individuals sampled in North Atlantic areas. Individuals are ordered by sampling area. Sampling-area abbreviations are shown on the first level below the plot, while the corresponding a priori ICCAT management stocks are shown on the second level. The vertical black line indicates the boundary between ATL-N and ATL-S. Sampling-area abbreviations: AZ, Azorean Islands; CI, Canary Islands; EA, Equatorial Atlantic; NS, Nova Scotia; PT, Portugal; VZ, Venezuela; WS, Western Sahara; GG, Gulf of Guinea; RGS, Rio Grande do Sul.

**Table 1:** Sampling areas included in the initial swordfish collection, grouped according to the three a priori ICCAT management stocks. Latitude and longitude indicate the geographic range of sampling positions within each area.

Sampling Area	Latitude	Longitude	Sampling Period	No. of Samples
**North Atlantic**				
Nova Scotia	41–45° N	52–66° W	July–October 2018; July–November 2019	63
Venezuela	8–14° N	50–67° W	July–December 2019; February–June 2020	22
Azorean Islands	35–40° N	19–32° W	May–June 2018	51
Portugal	35–38° N	7–18° W	August–December 2018; March 2019	43
Canary Islands	26–35° N	12–24° W	January–March 2018; May 2019	50
Western Sahara	22–33° N	23–13° W	June–July 2019	65
**South Atlantic**				
Rio Grande do Sul	33–36° S	49–51° W	October–November 2019	11
Equatorial Atlantic	2° S–5° N	0–22° W	May–August 2018; February–June 2019	81
Gulf of Guinea	0–2° S	0–8° E	July–August 2018	16
**Mediterranean**				
North Balearic Islands	38–42° N	0–3° E	April–December 2018	48
South Balearic Islands	37–39° N	0–3° E	June–July 2018	32
Ligurian Sea	43–44° N	7–9° E	August–September 2016; June–September 2018	25
Strait of Sicily	34–37° N	13–16° E	September 2019	31
Greece	35–36° N	25–26° E	June–November 2019	37

**Table 2:** Genetic-diversity statistics among the three a priori ICCAT management stocks. Estimates were calculated from the deduplicated, non-HWE-filtered 504-individual × 26,324-SNP dataset. Allelic richness (AR) was standardized by rarefaction to the smallest stock sample size (*n* = 103).

Stock	Ho	Hs	FIS	Mean AR
ATL-N	0.229	0.239	0.042	1.996
ATL-S	0.225	0.233	0.034	1.978
MED	0.236	0.243	0.029	1.903

Abbreviations: ATL-N, North Atlantic; ATL-S, South Atlantic; MED, Mediterranean; Ho, observed heterozygosity; Hs, within-population gene diversity; FIS, inbreeding coefficient; AR, allelic richness.

**Table 3:** Genetic-diversity statistics among the 14 sampling areas after excluding the 18 focal individuals with discordant or ambiguous assignments. Estimates were calculated from the deduplicated, non-HWE-filtered SNP panel; allelic richness (AR) was rarefied to 10 individuals, corresponding to the smallest sampling-area sample size.

Sampling Area	Ho	Hs	FIS	Mean AR
**North Atlantic (ATL-N)**				
Nova Scotia	0.227	0.235	0.035	1.619
Azorean Islands	0.228	0.236	0.035	1.622
Western Sahara	0.231	0.238	0.026	1.625
Canary Islands	0.228	0.237	0.036	1.623
Portugal	0.227	0.236	0.039	1.622
Venezuela	0.225	0.235	0.042	1.618
**South Atlantic (ATL-S)**				
Gulf of Guinea	0.223	0.233	0.041	1.611
Equatorial Atlantic	0.225	0.232	0.031	1.611
Rio Grande do Sul	0.223	0.230	0.032	1.603
**Mediterranean (MED)**				
North Balearic Islands	0.235	0.243	0.031	1.611
South Balearic Islands	0.235	0.243	0.030	1.611
Ligurian Sea	0.238	0.243	0.019	1.611
Strait of Sicily	0.236	0.242	0.026	1.609
Greece	0.234	0.242	0.032	1.608

Abbreviations: ATL-N, North Atlantic; ATL-S, South Atlantic; MED, Mediterranean; Ho, observed heterozygosity; Hs, within-population gene diversity; FIS, inbreeding coefficient; AR, allelic richness.

**Table 4:** Pairwise FST estimates among the 14 swordfish sampling areas after exclusion of the 18 focal individuals with discordant or ambiguous assignments. Estimates were calculated from the LD-pruned 15,049-SNP dataset. Significant comparisons (*p* < 0.05; 100 locus-bootstrap replicates) are shown in bold.

Area	NS	AZ	WS	CI	PT	VZ	GG	EA	RGS	NBI	SBI	LS	SoS	GR
Nova Scotia														
Azorean Islands	**0.0004**													
Western Sahara	**0.0014**	**0.0003**												
Canary Islands	**0.0013**	0.0003	**0.0003**											
Portugal	**0.0013**	**0.0004**	0.0000	**0.0006**										
Venezuela	**0.0005**	−0.0001	**0.0007**	**0.0006**	**0.0006**									
Gulf of Guinea	**0.0117**	**0.0079**	**0.0057**	**0.0069**	**0.0062**	**0.0096**								
Equatorial Atlantic	**0.0124**	**0.0086**	**0.0059**	**0.0069**	**0.0071**	**0.0098**	−0.0001							
Rio Grande do Sul	**0.0167**	**0.0122**	**0.0098**	**0.0107**	**0.0105**	**0.0137**	**0.0018**	**0.0015**						
North Balearic Islands	**0.0822**	**0.0831**	**0.0838**	**0.0842**	**0.0843**	**0.0834**	**0.0993**	**0.0992**	**0.1052**					
South Balearic Islands	**0.0819**	**0.0828**	**0.0832**	**0.0838**	**0.0838**	**0.0834**	**0.0985**	**0.0985**	**0.1045**	0.0000				
Ligurian Sea	**0.0822**	**0.0829**	**0.0833**	**0.0838**	**0.0842**	**0.0837**	**0.0993**	**0.0989**	**0.1056**	0.0004	**0.0007**			
Strait of Sicily	**0.0823**	**0.0834**	**0.0840**	**0.0844**	**0.0845**	**0.0839**	**0.0996**	**0.0996**	**0.1060**	0.0002	−0.0001	**0.0012**		
Greece	**0.0832**	**0.0843**	**0.0848**	**0.0852**	**0.0853**	**0.0851**	**0.1003**	**0.1001**	**0.1068**	**0.0006**	**0.0005**	−0.0001	**0.0013**	

Abbreviations: NS, Nova Scotia; AZ, Azorean Islands; WS, Western Sahara; CI, Canary Islands; PT, Portugal; VZ, Venezuela; GG, Gulf of Guinea; EA, Equatorial Atlantic; RGS, Rio Grande do Sul; NBI, North Balearic Islands; SBI, South Balearic Islands; LS, Ligurian Sea; SoS, Strait of Sicily; GR, Greece.

**Table 5:** Genetic stock assignments and uncertainty metrics for the 18 focal swordfish individuals showing discordant or ambiguous assignments relative to their a priori ICCAT management stock. All individuals had a rubias posterior assignment probability (PofZ) of 1.00. The global ADMIXTURE K = 2 column reports the Mediterranean-associated ancestry component, whereas the Atlantic-only K = 2 column reports the South-Atlantic-associated ancestry component. DAPC values indicate posterior assignment probabilities and the corresponding assigned stock.

Sample	A Priori Stock	Rubias Assignment	z-Score Percentile	ADMIXTURE K = 2 MED	Atlantic-Only K = 2 ATL-S	DAPC
AM190038	ATL-N	MED	97.6	1	NA	>0.99 (MED)
SWOLP026	ATL-N	MED	97.6	1	NA	>0.99 (MED)
SWOLP029	ATL-N	MED	97.6	1	NA	>0.99 (MED)
AM190022	ATL-N	MED	85.4	1	NA	>0.99 (MED)
AM190030	ATL-N	MED	85.4	1	NA	>0.99 (MED)
DF190318	ATL-N	MED	97.6	1	NA	>0.99 (MED)
DF190329	ATL-N	MED	75.6	1	NA	>0.99 (MED)
DF190077	ATL-N	MED	65.9	1	NA	>0.99 (MED)
DF190240	ATL-N	MED	97.6	1	NA	>0.99 (MED)
DF190058	ATL-N	MED	90.2	1	NA	>0.99 (MED)
DF190059	ATL-N	MED	95.1	1	NA	>0.99 (MED)
CT183008	ATL-N	ATL-S	26.3	0.0032	0.771	>0.99 (ATL-S)
CT183040	ATL-N	ATL-S	69.7	0	0.808	>0.99 (ATL-S)
CT193310	ATL-N	ATL-S	0	0.0353	0.758	>0.99 (ATL-S)
SI190196	ATL-N	ATL-S	60.5	0.0015	0.883	>0.99 (ATL-S)
SI190245	ATL-N	ATL-S	96.1	0	0.899	>0.99 (ATL-S)
SI190678	ATL-N	ATL-S	39.5	0.0053	0.59	0.73 (ATL-S)
ML190057	ATL-S	ATL-N	58.7	0.0302	0.207	0.99 (ATL-N)

Abbreviations: ATL-N, North Atlantic; ATL-S, South Atlantic; MED, Mediterranean; DAPC, discriminant analysis of principal components; NA, not applicable.

## Data Availability

The swordfish reference genome, associated structural and functional gene annotation files, and the VCF dataset generated in this study are available through Mendeley Data (https://doi.org/10.17632/c4nwbwbdbc.1). Raw ddRAD sequencing reads are not included in the repository.

## Supplementary Materials

The following supporting information can be downloaded at https://www.mdpi.com/article/10.3390/ijms27198612/s1. References [52,53,54,55,56,57,58,59,60,61,62,63,64,65,66,67,68] are cited in the supplementary materials.
