# Supplementary material for: Population Genomics Refines Population Structure and Individual Stock Assignment in Atlantic and Mediterranean Swordfish (Xiphias gladius)
Source: Int J Mol Sci. 2026 Sep 26;27(19):8612. doi: 10.3390/ijms27198612 (PMC13665000; doi:10.3390/ijms27198612)
Supplement: Supplementary file 1 [file ijms-27-08612-s001.zip › ijms-4491921-supplementary.pdf]

Supplementary Materials

International Journal of Molecular Sciences

Supplementary Methods S1. Reference-genome generation and annotation

S1.1. DNA extraction and sequencing

Blood DNA was extracted using the HMW DNA Extraction Kit following the manufacturer's protocol. DNA concentration and integrity were assessed using a NanoPhotometer P-Class (Implen, Munich, Germany) and GelRed staining on a 1% agarose gel. A hybrid sequencing strategy combining Oxford Nanopore MinION long reads and Illumina NovaSeq 6000 paired-end reads was used. The Nanopore and Illumina datasets provided approximately 20× and 30× genomic coverage, respectively.

S1.2. Genome assembly, polishing and chromosome anchoring

Illumina paired-end reads were quality assessed with FastQC [52] and processed with BBDuk [33] to remove low-quality bases (Phred < 30), adapters and reads shorter than 35 bp. Genome size was estimated from high-quality reads using BBMap [33]. Nanopore reads were corrected with NECAT [53] and independently assembled with Canu [26] and wtdbg2 [27] using an expected genome size of 600 Mb. The raw assemblies were corrected with three iterations of NTHITS (-b 36 -k 40 -t 36 --outbloom --solid) and NTEDIT [54] (-k 40 -z 1000 -d 3), merged with Quickmerge [55] (-l 490000), and subjected to five additional NTHITS/NTEDIT [54] polishing iterations.

Illumina reads were mapped to the merged assembly with minimap2 [34]. Platypus [36] was used to identify mismatches using the following settings: --minReads=6, --nCPU=20, --minFlank=10, --trimReadFlank=10, --assemble=1, --assembleBadReads=1, --assemblerKmerSize=55, --assembleBrokenPairs=1, --minMapQual=30, --minBaseQual=30 and --minPosterior=30. High-quality homozygous PASS variants were incorporated with vcf-consensus [37].

Published swordfish RNA-seq reads [31] were mapped to the assembly with minimap2 [34] using the -x splice option. The resulting mapping file was processed with Opossum [56] (--MinFlankEnd 10, --MinFlankStart 10, --SoftClipsExist True and --ProperlyPaired False) and used for two additional Pilon [57] polishing iterations (--minqual 30, --minmq 30, --mindepth 6, --flank 0, --threads 20, --duplicates, --fix bases, --diploid and --changes). Assembly statistics were calculated with QUAST [28]. Final scaffolds were anchored to the chromosome-scale swordfish reference assembly [29] using RaGOO [30] with default parameters. For downstream analyses, unanchored scaffolds were retained as separate scaffolds rather than treated as a true chromosome.

S1.3. Structural and functional annotation

Repetitive elements were annotated with RepeatMasker [58]. Published swordfish RNA-seq reads [31] were mapped to the genome with STAR [59] in two-pass mode and assembled genome-guided with Trinity v2.8.6 [60] using --SS_lib_type RF, --jaccard_clip, --full_cleanup, --min_kmer_cov 2, --no_normalize_reads and --genome_guided_max_intron 100000. Only transcripts longer than 500 bp were retained.

An initial MAKER [61] annotation integrated the RepeatMasker output, the Trinity transcriptome and the zebrafish gene model implemented in AUGUSTUS [62]. High-confidence swordfish gene models from this initial run were used to train a swordfish-specific AUGUSTUS model with etraining and optimize_augustus.pl. STAR alignments were also used with GeneMark [63] to generate an HMM model for gene prediction. A final MAKER run integrated transcript evidence, repeat annotation, the swordfish-specific AUGUSTUS [62] model, the GeneMark [63] model and protein evidence from Lates calcarifer (ASM164080v1).

The resulting GFF3 annotation was converted to GTF with gffread [64]. StringTie [65] was applied to the RNA-seq alignments to refine isoforms and identify additional non-coding transcripts, and GenomeTools [66] was used for final feature formatting. Putative long non-coding RNAs were classified with PLEK [67]. Gene descriptions and Gene Ontology annotations were assigned with PANNZER2 [68] using minimum query or subject coverage of 0.4 and a minimum alignment length of 50 amino acids. KEGG annotation was performed using the reference-organism set hsa, mmu, rno, dre, cel, ecu, nme, bsu, mtu, syn, aae, ape, lcf, npr, lcm, sdu, tru, ipu, srx, xma, onl and cvg.

S1.4. Genome completeness assessment

Genome completeness was assessed with BUSCO [32] using the Actinopterygii, Metazoa and Eukaryota lineage datasets. The Actinopterygii lineage dataset was considered the primary assessment because of its greater taxonomic

specificity for swordfish, whereas the broader Metazoa and Eukaryota datasets were used as complementary assessments. Complete BUSCO [32] results are reported in Table S1.

Supplementary Tables

Table S1. BUSCO assessment of swordfish reference-genome completeness using the Actinopterygii, Metazoa and Eukaryota lineage datasets. Values indicate the number and percentage of complete single-copy, complete duplicated, fragmented and missing orthologues.

BUSCO lineage	Complete single-copy, n (%)	Complete duplicated, n (%)	Fragmented, n (%)	Missing, n (%)	Complete total (%)
Actinopterygii	3354 (92.1)	44 (1.2)	65 (1.8)	177 (4.9)	93.4
Metazoa	883 (92.6)	24 (2.5)	13 (1.4)	34 (3.6)	95.1
Eukaryota	223 (87.5)	4 (1.6)	7 (2.7)	21 (8.2)	89.0

Table S2. Chromosome-level assembly and annotation summary for the swordfish reference genome. The table reports the number of anchored scaffolds and annotated genes for each chromosome, together with chromosome 0 and unanchored scaffolds.

Chromosome	Anchored scaffolds	Number of genes
1	151	1930
2	154	1378
3	137	1980
4	174	1705
5	157	1380
6	182	1978
7	139	1696
8	204	2248
9	129	1640
10	178	1786
11	165	1607
12	131	1356
13	65	776
14	124	1781
15	185	2477
16	155	1712
17	191	1899
18	200	2102
19	225	2120
20	149	1686
21	183	2203
22	148	2237
23	169	1658
24	115	1784
0	148	52
Unanchored scaffolds	535	1058

Table S3. Sampling-area accounting from the initial swordfish collection to the final analytical dataset. "Removed overall" includes individuals excluded during initial quality filtering and those subsequently removed following duplicate/sample-identity assessment.

**Sampling area**	**Initial n**	**Final retained n**	**Removed overall**	**Removed overall (%)**
Nova Scotia	63	57	6	9.5
Venezuela	22	20	2	9.1
Azorean Islands	51	44	7	13.7
Portugal	43	38	5	11.6
Canary Islands	50	41	9	18.0
Western Sahara	65	58	7	10.8
Rio Grande do Sul	11	10	1	9.1
Equatorial Atlantic	81	80	1	1.2
Gulf of Guinea	16	16	0	0.0
North Balearic Islands	48	41	7	14.6
South Balearic Islands	32	24	8	25.0
Ligurian Sea	25	20	5	20.0
Strait of Sicily	31	23	8	25.8
Greece	37	32	5	13.5
Total	575	504	71	12.3

Table S4. Distribution of sequencing depth, genotype missingness and SNP call rate among the 504 retained swordfish individuals, the three a priori ICCAT management stocks and the 14 sampling areas. Values are reported as median (interquartile range, IQR).

Group	n	Read depth median (IQR)	Missing-genotype rate (%) median (IQR)	SNP call rate (%) median (IQR)
All retained individuals	504	11.48 (8.01–16.42)	0.84 (0.26–2.50)	99.16 (97.50–99.74)
ATL-N	261	12.78 (8.80–17.73)	0.70 (0.23–1.99)	99.30 (98.01–99.77)
ATL-S	103	9.67 (7.27–15.28)	0.57 (0.22–1.46)	99.43 (98.54–99.78)
MED	140	10.07 (7.53–14.05)	1.59 (0.70–4.39)	98.41 (95.61–99.30)
Nova Scotia	57	12.78 (9.86–17.02)	0.72 (0.26–2.01)	99.28 (97.99–99.74)
Azorean Islands	44	11.56 (7.99–14.33)	0.88 (0.38–2.22)	99.12 (97.78–99.62)
Western Sahara	58	11.30 (7.93–14.65)	0.31 (0.17–1.46)	99.69 (98.54–99.83)
Canary Islands	41	14.22 (8.57–19.59)	0.84 (0.33–4.89)	99.16 (95.11–99.67)
Portugal	38	17.60 (12.84–20.57)	0.51 (0.21–0.97)	99.49 (99.03–99.79)
Venezuela	20	15.01 (8.04–23.31)	1.51 (0.59–3.08)	98.49 (96.92–99.41)
Gulf of Guinea	16	16.77 (7.31–20.98)	0.48 (0.22–3.04)	99.52 (96.96–99.78)
Equatorial Atlantic	80	9.64 (7.46–14.66)	0.60 (0.22–1.25)	99.40 (98.75–99.78)
Rio Grande do Sul	10	7.29 (6.20–9.08)	0.81 (0.38–2.05)	99.19 (97.95–99.62)
North Balearic Islands	41	11.97 (8.93–18.34)	1.21 (0.35–3.24)	98.79 (96.76–99.65)
South Balearic Islands	24	12.30 (8.52–21.59)	0.96 (0.23–3.64)	99.04 (96.36–99.77)
Ligurian Sea	20	7.40 (6.84–8.57)	4.85 (1.63–8.31)	95.15 (91.69–98.37)
Strait of Sicily	23	13.07 (8.13–15.77)	1.32 (0.71–2.25)	98.68 (97.75–99.29)
Greece	32	10.08 (7.67–11.97)	2.28 (1.50–4.01)	97.72 (95.99–98.50)

Note: Read depth and missing-genotype rate were obtained from the per-individual quality-control dataset for the 504 retained individuals. SNP call rate was calculated as 1 − missing-genotype rate. ATL-N, North Atlantic; ATL-S, South Atlantic; MED, Mediterranean.

Table S5. Sampling-area genetic-diversity statistics calculated before exclusion of the 18 focal individuals with discordant or ambiguous genetic assignments. Ho, observed heterozygosity; Hs, expected gene diversity; FIS, inbreeding coefficient; Total AR, total rarefied allelic richness; Mean AR, mean rarefied allelic richness per locus.

Sampling area	Ho	Hs	FIS	Total AR	Mean AR
North Atlantic (ATL-N)					
Nova Scotia	0.227	0.235	0.035	42,625.33	1.619
Azorean Islands	0.228	0.236	0.035	42,707.61	1.622
Western Sahara	0.231	0.237	0.027	42,763.16	1.624
Canary Islands	0.229	0.243	0.058	43,251.37	1.643
Portugal	0.228	0.244	0.064	43,348.62	1.647
Venezuela	0.225	0.235	0.042	42,590.15	1.618
South Atlantic (ATL-S)					
Gulf of Guinea	0.223	0.233	0.041	42,406.57	1.611
Equatorial Atlantic	0.227	0.233	0.027	42,444.10	1.612
Rio Grande do Sul	0.223	0.230	0.032	42,204.32	1.603
Mediterranean (MED)					
North Balearic Islands	0.235	0.243	0.031	42,408.51	1.611
South Balearic Islands	0.235	0.243	0.030	42,399.40	1.611
Ligurian Sea	0.238	0.243	0.019	42,409.27	1.611
Strait of Sicily	0.236	0.242	0.026	42,347.55	1.609
Greece	0.234	0.242	0.032	42,335.60	1.608

Table S6. Pairwise FST estimates among the 14 swordfish sampling areas calculated using the complete 504-individual dataset and the a priori ICCAT stock classification. Significant comparisons ($P < 0.05$; 100 locus-bootstrap replicates) are shown in bold.

	NS	AZ	WS	CI	PT	VZ	GG	EA	RGS	NBI	SBI	LS	SoS	GR
NS														
AZ	0.0004													
WS	0.0017	0.0004												
CI	0.0020	0.0012	0.0015											
PT	0.0027	0.0021	0.0021	0.0002										
VZ	0.0005	-0.0001	0.0008	0.0011	0.0019									
GG	0.0117	0.0079	0.0052	0.0087	0.0093	0.0096								
EA	0.0121	0.0083	0.0053	0.0091	0.0104	0.0096	0.0000							
RGS	0.0167	0.0122	0.0092	0.0125	0.0131	0.0137	0.0018	0.0015						
NBI	0.0822	0.0831	0.0844	0.0660	0.0608	0.0834	0.0993	0.0989	0.1052					
SBI	0.0819	0.0828	0.0837	0.0654	0.0600	0.0834	0.0985	0.0981	0.1045	0.0000				
LS	0.0822	0.0829	0.0838	0.0651	0.0603	0.0837	0.0993	0.0986	0.1056	0.0004	0.0007			
SoS	0.0823	0.0834	0.0845	0.0659	0.0607	0.0839	0.0996	0.0993	0.1060	0.0002	-0.0001	0.0012		
GR	0.0832	0.0843	0.0853	0.0667	0.0616	0.0851	0.1003	0.0998	0.1068	0.0006	0.0005	-0.0001	0.0013	

Abbreviations: NS, Nova Scotia; AZ, Azorean Islands; WS, Western Sahara; CI, Canary Islands; PT, Portugal; VZ, Venezuela; GG, Gulf of Guinea; EA, Equatorial Atlantic; RGS, Rio Grande do Sul; NBI, North Balearic Islands; SBI, South Balearic Islands; LS, Ligurian Sea; SoS, Strait of Sicily; GR, Greece.

Table S7. Global ADMIXTURE K-selection results for the complete 504-individual dataset. Mean log-likelihood and its standard deviation summarize reproducibility across independently seeded replicates, whereas mean 10-fold cross-validation (CV) error and its standard deviation summarize predictive performance across K = 1–6.

K	Mean log-likelihood	SD log-likelihood	Mean CV error	SD CV error
1	−5,470,319	0 (exact)	0.4617	0.000011
2	−5,399,984	0.003	0.42500	0.000015
3	−5,383,804	30.8	0.42426	0.000015
4	−5,371,760	236.4	0.4263	0.00078
5	−5,360,972	296.9	0.4283	0.00064
6	−5,352,106	366.6	0.4305	0.00050

Table S8. ADMIXTURE cross-validation results for the complete unbalanced dataset and for ten independently generated sample-size-balanced subsamples. Values for the balanced dataset are means across the ten subsamples and two cross-validation replicates per subsample.

K	CV error, raw (unbalanced) dataset	CV error, balanced dataset (mean of 10 subsamples × 2 replicates)
1	0.4617	0.4644
2	0.42500	0.42436
3	0.42426	0.42464

Table S9. ADMIXTURE K-selection results for the Atlantic-only subset (n = 353; 250 ATL-N and 103 ATL-S). Mean log-likelihood and its standard deviation summarize reproducibility across independently seeded replicates, whereas mean 10-fold cross-validation (CV) error was used to compare K = 1–4.

K	Mean log-likelihood	Mean CV error	SD log-likelihood
1	-3,861,928	0.43248	0 (exact)
2	-3,837,783	0.43139	0.00001 (near-exact)
3	-3,826,832	0.43357	106.3
4	-3,816,414	0.43673	193.4

Table S10. Convergence and sensitivity diagnostics for the rubias Bayesian genetic-stock-identification analysis.

Diagnostic	Result
Effective sample size of post-burn-in mixing proportions	1,827–2,244 out of 2,000 sweeps
Geweke z-score across chains and repunits	-1.81 to +1.81
Cross-chain agreement in mixing proportions	within 0.005
Extended MCMC sensitivity run	max |ΔPofZ| = 1.1 × 10−13; max |Δz-score| = 0; identical top assignment for 18/18 focal individuals

Note: The extended sensitivity run used 50,000 repetitions with a burn-in of 10,000, compared with 2,000 repetitions and a burn-in of 200 in the original analysis.

Supplementary Figure

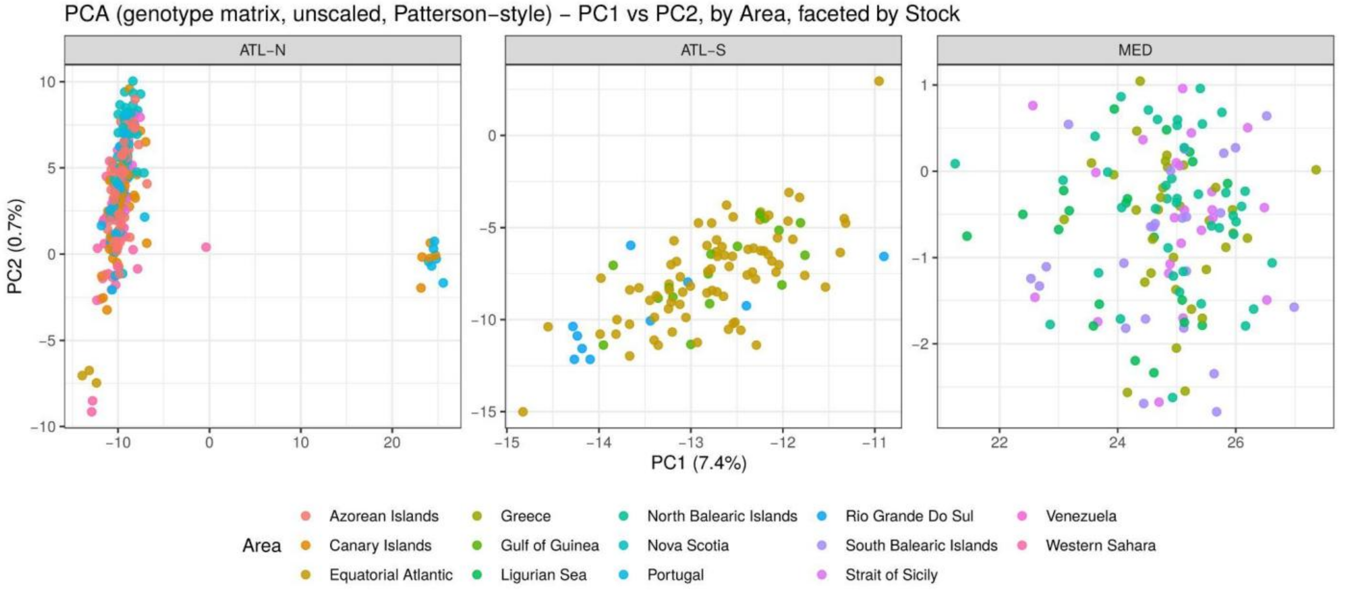

Figure S1. Unscaled genotype-matrix principal component analysis (PCA) of 504 swordfish based on the LD-pruned 15,049-SNP dataset, coloured by sampling area and faceted according to the three a priori ICCAT management stocks (ATL-N, ATL-S and MED). PC1 and PC2 explain 7.4% and 0.7% of total genetic variance, respectively. Sampling areas showed extensive overlap within each management stock, with no distinct area-level subclusters.
